# Author Correction: Influence of microstructure on superconductivity in K_*x*_Fe_2−*y*_Se_2_ and evidence for a new parent phase K_2_Fe_7_Se_8_

**DOI:** 10.1038/s41467-020-14681-7

**Published:** 2020-02-21

**Authors:** Xiaxin Ding, Delong Fang, Zhenyu Wang, Huan Yang, Jianzhong Liu, Qiang Deng, Guobin Ma, Chong Meng, Yuhui Hu, Hai-Hu Wen

**Affiliations:** 1 National Laboratory of Solid State Microstructures and Department of Physics, National Center of Microstructures and Quantum Manipulation, Nanjing University, Nanjing, 210093 China; 2 National Laboratory for Superconductivity, Institute of Physics and National Laboratory for Condensed Matter Physics, Chinese Academy of Sciences, Beijing, 100190 China

Correction to: *Nature Communications* 10.1038/ncomms2913, published online 21 May 2013

In light of concerns that have been raised regarding the measurement, analysis, and presentation of the sample compositions from energy-dispersive X-ray spectra (EDS) in Fig. 4, the authors would like to add a number of clarifications and amendments to the published version of this Article:

In the original version of this Article, the 13th, 14th, 15th, and 16th sentences of the first paragraph of the ‘Scanning electron microscope measurements’ section of the Results currently read ‘As presented in Fig. 4a, b, close correlation between the local compositions of K and Fe is observed. By figuring out the local compositions, we find that the brighter domains have a composition of about K_0.64_Fe_1.78_Se_2_, while the background area has a composition of about K_0.81_Fe_1.60_Se_2_, the latter is very close to the standard K_2_Fe_4_Se_5_ phase. To strengthen these conclusions, we did the local analysis on 50 randomly selected specific points, marked by the red spots (on background) and blue spots (on the domains) in Fig. 4c. The statistics on these data are given in Fig. 4d. Interestingly, the data are rather converged and fall into mainly two groups, one with the composition of about K_0.68_Fe_1.78_Se_2_ and another one around K_0.8_Fe_1.63_Se_2_.’

We apologize that these sentences are imprecise. The quantitative numbers in Fig. 4b cannot be obtained from the line scan profile data. Therefore, the four horizontal lines in Fig. 4b should be ignored. We have also added to this panel the spatial distribution of Se composition along the same line. We have also revised Fig. 4d to use all 79 data points to analyze the compositions. Therefore, the averaged compositions after the EDS analysis have been corrected from K_0.68_Fe_1.78_Se_2_ to K_0.69_Fe_1.78_Se_2_ for domains, and from K_0.80_Fe_1.63_Se_2_ to K_0.80_Fe_1.65_Se_2_ in background areas.

The correct version of these sentences should read ‘As presented in Fig. 4a, b, close correlation between the local compositions of K and Fe is observed; that of Se is relatively more uniform. Although we did the line scan measurements of compositions of K and Fe following the yellow arrowed line in Fig. 4a, it is known that the line scan measurements usually cannot tell precise compositions. To get more accurate compositions, we did the local analysis on 79 selected points, among them 39 points are on the domains and 40 in the background region. The statistics on these data are given in Fig. 4d. Interestingly, the data are rather converged and fall into mainly two groups, one with the average composition of about K_0.69_Fe_1.78_Se_2_ and another one around K_0.8_Fe_1.65_Se_2_.’

The last sentence of this same paragraph reads ‘Further analysis on the sample S350 gives the similar compositions in two different regions as the SFC.’ We apologize for the imprecise description of this in the paper. We did not include any EDS data for sample S350 in Fig. 4. The correct version of this sentence should read ‘Further analysis based on grey-scale and XRD measurements suggest that the sample S350 may have similar compositions in the two different regions as the SFC, which is detailed below.’

In light of the above errors, the corrected version of Fig. 4 and its caption are:

**Fig. 4:**
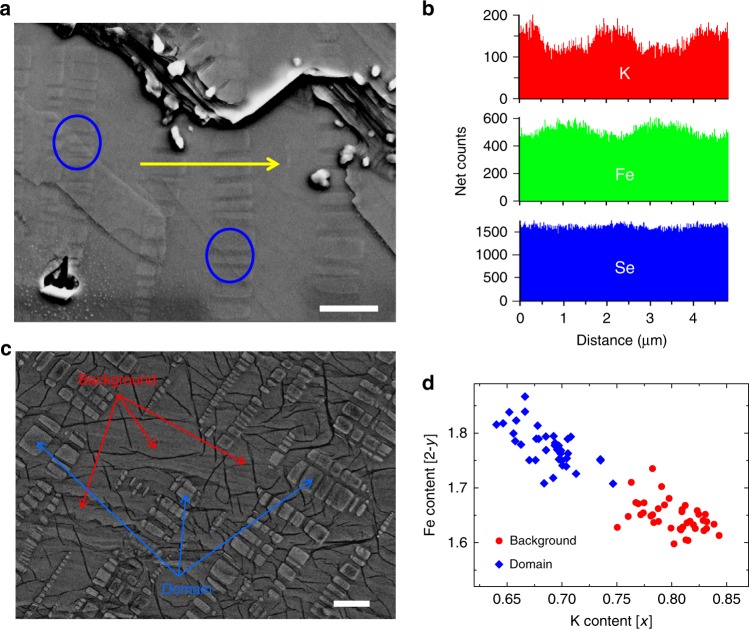
Correlations between the microstructure and the analysis on the compositions. **a** The topography of one cleaved surface of a sample SFC. Scale bar, 2 μm. One can clearly see the rectangular domains that are buried in the background. The yellow arrowed line highlights the trace along which the line scan profile of the compositions was taken, as shown in (**b**). The large blue circles here mark the positions where the rectangular domains go through several layers along the *c* axis. The outline and shade of the domains can be seen on the layers with different height. **c** Representative SEM image (taken with an accelerating voltage of 10 kV) of the typical domain (marked by blue arrows) and background (marked by red arrows) regions. From these two different regions, the EDS spectra were collected for pointwise composition analysis. Scale bar, 2 μm. **d** The compositions of K and Fe were measured from the total 79 spots on the domain and on the background regions of three SFC samples cleaved from a single bulk. One can clearly see that the EDS data fall into two groups. By analyzing the total EDS dataset (39 points on the domains and 40 points in the background region), we get the averaged compositions with the formula K_0.69_Fe_1.78_Se_2_ on the domain, and K_0.80_Fe_1.65_Se_2_ on the background, respectively. The measurements are done with the voltage of 25 kV.

Specifically, the fourth sentence in the original legend of Fig. 4 currently reads ‘The yellow arrowed line highlights the trace along which the spatial distribution of compositions of K and Fe are measured and presented in (**b**).’ In the corrected version of the legend, this sentence reads ‘The yellow arrowed line highlights the trace along which the line scan profile of the compositions was taken, as shown in (**b**).’

The seventh and eighth sentences of the original legend read ‘(**c**) The SEM image of the sample SFC in another region. The red spots and blue spots mark the positions where the local compositions are analysed.’ This is incorrect: these 25 red spots and 25 blue spots do not represent the precise locations from which EDS data were collected. In the corrected version of the legend, these two sentences state ‘(**c**) Representative SEM image (taken with an accelerating voltage of 10 kV) of the typical domain (marked by blue arrows) and background (marked by red arrows) regions. From these two different regions the EDS spectra were collected for point-wise composition analysis.’

The 10th and 11th sentences of the original legend read ‘(**d**) The compositions of K and Fe measured on the rectangular domains (blue diamond) and the background (red circles). One can clearly see that the data fall into two groups: the formula K_0.68_Fe_1.78_Se_2_ on the domain, and K_0.81_Fe_1.6_Se_2_ on the background.’ In the original version of Fig. 4d, we presented the composition obtained from 50 different EDS data (25 from domains and 25 from background regions) that were selected from a total of 79 pointwise EDS measurements (40 on the background and 39 on the domains) collected from three samples cleaved from a single bulk SFC sample, instead of one sample indicated in the original paper. We want to clarify that there is no positionwise correlation between these data and those points marked in Fig. 4c of the original paper. We apologize for not presenting the full set of data points in the paper, which is a clear deviation of standard practice. The corrected version of the legend replaces these two sentences with ‘(**d**) The compositions of K and Fe were measured from the total 79 spots on the domain and on the background regions of three SFC samples cleaved from a single bulk. One can clearly see that the EDS data fall into two groups. By analyzing the total EDS dataset (39 points on the domains and 40 points in the background region), we get the average compositions with the formula K_0.69_Fe_1.78_Se_2_ on the domain, and K_0.80_Fe_1.65_Se_2_ on the background, respectively.’

The final sentence of the original legend reads ‘The measurements are done with the voltage of 20 kV.’ The corrected version states ‘25 kV’ instead of ‘20 kV’.

In addition to these errors in Fig. 4 and the related discussion, the fourth sentence in the third paragraph of the ‘Scanning electron microscope measurements’ section of the Results currently reads: ‘Similar behaviour of the composition distribution is observed in the sample S350, which presents better global appearance of superconductivity, although now the domains become much smaller.’ This is incorrect. This sentence should be replaced with: ‘Similar grey-scale fraction is observed in samples SFC and S350, which may suggest similar compositions of the superconducting areas of SFC and S350, the latter presents better global appearance of superconductivity, although now the domains become much smaller.’

The first sentence of the Discussion reads ‘In our local analysis presented above, it is found that the composition of the superconducting domains are converged at about K_0.68_Fe_1.78_Se_2_.’ It should say ‘K_0.69_Fe_1.78_Se_2_’ instead of ‘K_0.68_Fe_1.78_Se_2_’.

The fourth and fifth sentences of the Discussion read ‘Concerning the uncertainties in these totally distinct techniques, the consistency between the two techniques are remarkable, which also validates the argument that the domains are superconducting and have a composition of about K_0.68_Fe_1.78_Se_2_. In the background phase, the analysis shows a composition of K_0.8_Fe_1.63_Se_2_, which is naturally attributed to the well-known 245 phase.’ These should say ‘K_0.69_Fe_1.78_Se_2_’ instead of ‘K_0.68_Fe_1.78_Se_2_’ and ‘K_0.8_Fe_1.65_Se_2_’ in place of ‘K_0.8_Fe_1.63_Se_2_’.

The 17th sentence of the same paragraph reads ‘A glance at our formula K_0.68_Fe_1.78_Se_2_ of the superconducting domains would suggest that a possible phase with the formula of K_0.5_Fe_1.75_Se_2_ (or called as 278 phase) is next to the K_0.8_Fe_1.6_Se_2_ (or the 245) phase and may act as the parent phase for superconductivity.’ It should say ‘K_0.69_Fe_1.78_Se_2_’ instead of ‘K_0.68_Fe_1.78_Se_2_’.

The 12th sentence of the second paragraph in the Discussion reads ‘Although our STM data here cannot give a direct evidence for the 1/8 Fe-vacancy $\sqrt 8 \times \sqrt {10}$ state, the expectation of the composition of this state is very consistent with the chemical composition obtained from the superconducting domains, that is, K_0.68_Fe_1.78_Se_2_.’ It should say ‘K_0.69_Fe_1.78_Se_2_’ instead of ‘K_0.68_Fe_1.78_Se_2_’.

The fifth sentence of the ‘Structure characterization’ section of the Methods currently reads ‘In the spot mode, 50 spots were randomly selected on and off the domains of each sample to obtain high resolution spectra with a measurement time of 1 min for one point, with the voltage of 20 kV.’ This should state ‘79 spots were selected’ instead of ‘50 spots were randomly selected’, ‘of three SFC samples’ instead of ‘of each sample’ and ‘25 kV’ instead of ‘20 kV’.

Finally, the third sentence of the Author Contributions section originally reads ‘The SEM measurements and analysis were carried out by X.D., G.M., C.M. and Y.H.’ This should instead read ‘The SEM and line scan measurements were carried out by X.D., G.M. and C.M.; X.D. and Y.H. did the analysis of the EDS data and made their connection to the SEM measurements.’

These errors have not been corrected in the PDF or HTML versions of the Article.

